# Metagenome-assembled genomes provide insight into the microbial taxonomy and ecology of the Buhera soda pans, Zimbabwe

**DOI:** 10.1371/journal.pone.0299620

**Published:** 2024-12-02

**Authors:** Ngonidzashe Mangoma, Nerve Zhou, Thembekile Ncube

**Affiliations:** 1 Faculty of Applied Science, Department of Applied Biology and Biochemistry, National University of Science and Technology, Bulawayo, Zimbabwe; 2 Faculty of Sciences, Biological Sciences and Biotechnology Department, Botswana International University of Science and Technology, Palapye, Botswana; 3 Research and Internationalisation Office, National University of Science and Technology, Bulawayo, Zimbabwe; Universidade Estadual de Ponta Grossa, BRAZIL

## Abstract

The use of metagenomics has substantially improved our understanding of the taxonomy, phylogeny and ecology of extreme environment microbiomes. Advances in bioinformatics now permit the reconstruction of almost intact microbial genomes, called metagenome-assembled genomes (MAGs), from metagenomic sequence data, allowing for more precise cell-level taxonomic, phylogenetic and functional profiling of uncultured extremophiles. Here, we report on the recovery and characterisation of metagenome-assembled genomes from the Buhera soda pans located in eastern Zimbabwe. This ecosystem has not been studied despite its unique geochemistry and potential as a habitat for unique microorganisms. Metagenomic DNA from the soda pan was sequenced using the DNA Nanoball Sequencing (DNBSEQ^R^) technique. Sequence analysis, done on the Knowledgebase (KBase) platform, involved quality assessment, read assembly, contig binning, and MAG extraction. The MAGs were subjected to taxonomic placement, phylogenetic profiling and functional annotation in order to establish their possible ecological roles in the soda pan ecosystem. A total of 16 bacterial MAGs of medium to high quality were recovered, all distributed among five phyla dominated by *Pseudomonadota* and *Bacillota*. Of the ten MAGs that were taxonomically classified up to genus level, five of them belonged to the halophilic/ haloalkaliphilic genera *Alkalibacterium*, *Vibrio*, *Thioalkalivibrio*, *Cecembia* and *Nitrincola*, underscoring the importance of haloalkaliphiles in the Buhera soda pans. Functional profiling revealed the possession of diverse carbohydrate-metabolising pathways by the MAGs, with glycolysis and the pentose phosphate pathways appearing to be key pathways in this ecosystem. Several MAGs possessed pathways that implicated them in some key aspects of the nitrogen and sulphur cycle. Some MAGs harboured both sulphate reduction and respiratory pathways, suggesting a possible mechanism of ATP biosynthesis through sulphate respiration. This study demonstrates the feasibility of the recovery and taxonomic and functional annotation of high quality microbial genomes from extreme environments, making it possible to establish the ecological roles and biotechnological potential of uncultured microorganisms.

## 1. Introduction

The Buhera soda pans are a unique, naturally occurring saline and alkaline aquatic system located in a remote part of the Buhera district found in Eastern Zimbabwe. No record exists at present regarding the scientific exploration of this potentially important extreme environment. However, in our recent exploration of this site, we established that the Buhera soda pans are highly alkaline (pH 8.74–11.03), moderately saline (2.94–7.55 mg/l) and belong to the carbonate type of soda pans and lakes [1]. The geochemistry of the Buhera soda pans is similar to that of several other soda pans and lakes from around the world [2–4]. In general, soda pans and lakes have alkaline pH in the range 8–11, and highly variable salinity with a lower threshold of around 1 mg/l [5]. The extreme and distinctive geochemistry of the Buhera soda pans exerts selection pressure on their microbiomes, often leading to the evolution of unique microbial communities. The composition of the microbial communities of soda pans and lakes, and the possibility of finding novel microorganisms with tangible biotechnological potential often attracts interest in these microbial habitats.

The extreme conditions existent in soda pans and lakes modify their microbial communities, leading to the dominance of extremophiles, with halophiles, alkaliphiles and haloalkaliphiles often dominating these microbial habitats [6]. The ability of different microbial taxa to thrive under the alkaline and saline conditions of soda pans stimulate interest among microbiologists about such microbial ecosystems, and elevate the possibility that some of the microorganisms that inhabit these environments have potentially useful applications. For instance, numerous studies have demonstrated that soda pans and lakes harbour a rich diversity of novel microbes, enzymes and unique microbial metabolites [4]. Enzymes such as amylases, proteases, lipases and cellulases have been extracted from haloalkaliphiles isolated from saline-alkaline systems such as soda pans, and some of these enzymes have been demonstrated to perform optimally under conditions of alkaline pH and elevated salinity [7]. Enzymes that retain their stability and high performance under alkaline pH and salinity have several potential applications in industrial processes such as leather tanning, starch saccharification and other food processes, detergent making and the bioremediation of saline and/ or alkaline effluents such as those from the fat/ oil and soap industry [8,9]. The potential benefits from the exploration of the microbial communities of soda pans and lakes provide impetus for more inquiry into their microbial communities. A combination of both culture-based and culture-independent techniques have helped expose the rich microbial communities of soda pans and lakes [8,10–12]. However, it is estimated that up to 99% of the microbial inhabitants of extreme environments are unculturable [13], leading to an increasing reliance on emerging omics-based techniques such as metagenomics in studies of extreme microbiomes.

Recent innovations in DNA extraction, sequencing and sequence analysis technologies have exponentially broadened the scope of metagenomic studies of extreme environments, leading to the exploration of many new extreme environments and the discovery of new microbial taxa. While most metagenomics studies allow for read-based community-level determination of microbial community composition and function, new developments now make it possible to perform more precise species-level metagenomic analysis using techniques such as metagenome-assembled genome (MAG) construction. A metagenome-assembled genome (MAG) is a hypothetical microbial genome created using contigs derived from the assembly of metagenomic sequence reads [14]. Consequently, each MAG represents a putative microbial genome, and may contain enough information to enable its taxonomic placement, phylogenetic profiling and functional annotation [15]. MAG extraction is an innovative technique that allows not only for metagenomic sequence analysis to be performed at the level of individual microbial species, allowing more accurate description of their taxonomy and ecological function, but also allows for the discovery and characterisation of previously uncultured microorganisms especially from extreme environments such as soda pans. Thus, being able to recreate MAGs from extreme environment metagenomic DNA sequence data provides a back-door channel through which we can explore the microbial community of an extreme environment, without the usual limitations imposed by the physico-chemical demands of extremophiles.

In this study, we report on efforts to construct and characterise metagenome-assembled genomes (MAGs) from metagenomic DNA obtained from the Buhera soda pans found in eastern Zimbabwe. Several parameters are used to characterise the integrity of a MAG, and one such key parameter is MAG completeness, which indirectly reflects the size of the MAG relative to the reference genome. While many studies report on MAGs with completeness in the 50–60% range [15], we report on the creation of several MAGs with completeness above 80%. The high degree of completeness enables one to then make a variety of highly accurate predictions about an organism’s taxonomy, phylogeny and physiology, making it possible to precisely allocate ecological functions to uncultured extremophiles.

## 2. Materials and methods

### 2.1 Metagenomic DNA data acquisition

#### 2.1.1 Sample collection

Water samples were collected from the soda pans located at Gombahari village in Buhera rural district (-19.26342, 31.80437) in the Manicaland province of eastern Zimbabwe. Samples were collected at the peak of the hot and dry season in October 2021. The grab sampling techniques was employed and a total of five samples amounting to 100 ml each was collected. The samples were chilled on ice and immediately transported to the laboratory where 50 ml portions of each sample were mixed to create one composite sample and frozen at -80°C. This is the sample that was used for metagenomic DNA extraction.

#### 2.1.2 Metagenomic DNA extraction and sequencing

Total metagenomic DNA was extracted from the sample using the ZymoBIOMICS DNA Miniprep kit (Zymo Research, CA, USA). The concentration and quality of the extracted DNA was determined using a fluorometer (Qubit Fluorometer, Invitrogen), after which 1 μg of metagenomic DNA was randomly fragmented using the Covaris instrument (Covaris Inc. Woburn, MA, USA). Fragments of size 200–400 bp were selected using an Agencourt AMPure XP-Medium kit. The fragments were end-repaired using T4 Polynucleotide Kinase (T4 PNK) in a 100 μl master mix containing 88 μl of 1x T4 DNA ligase buffer (New England Biolabs–NEB, Ipswich, MA, USA), 2 μl of 25 mM dNTP mix, 5 μl of 10 U/μl T4 PNK (NEB), 4 μl of 3 U/μl T4 DNA polymerase I (NEB), and 1 μl of 5 U/μl Klenow fragment of DNA polymerase I (NEB), with the reaction being incubated at room temperature for 30 minutes. Polyadenylation of the fragments was performed using 100 μl master mix comprising 90 μl of 1x NEB buffer 2 (NEB), 5 μl of 10 mM dATP, and 5 μl of 5 U/μl Exo-Minus Klenow DNA Polymerase (NEB) and left to incubate at 37°C for 30 minutes. A 1x solid-phase reversible immobilization (SPRI) clean-up was performed with an elution in 100 ul of 1x NEB ligation buffer (NEB). Afterwards, 2 μl of NEB DNA Quick Ligase (NEB) and 3 μl of indexed DNA adapters (Illumina, San Diego, CA, USA) were added and mixed thoroughly. The library was amplified using a TruSeq DNA HT Sample Preparation Kit (Illumina, San Diego, CA USA) following the TruSeq DNA Sample Preparation Guide (#15026486 Rev. C, July 2012). The PCR conditions were as follows: initial denaturation at 98°C for 30 seconds; 10 cycles of denaturation at 98°C for 10 seconds, annealing at 60°C for 30 seconds, and extension at 72°C for 30 seconds; and final extension at 72°C for 5 minutes. PCR products were purified by the Agencourt AMPure XP-Medium kit. The double stranded PCR products were heat denatured and circularized by the splint oligo sequence. The single strand circular DNA (ssCir DNA) fragments were formatted as the final library. The qualified libraries were sequenced by the BGISEQ-500 process in which an ssCir (single-stranded circular) DNA molecule forms a DNA nanoball (DNB) containing more than 300 copies through rolling-cycle replication. The DNBs were loaded into the patterned nano array using high density DNA nanochip technology. Finally, paired-end reads of 100 bp size were obtained by combinatorial Probe-Anchor Synthesis (cPAS).

### 2.2 Metagenomic sequence analysis

**Fig 1:**
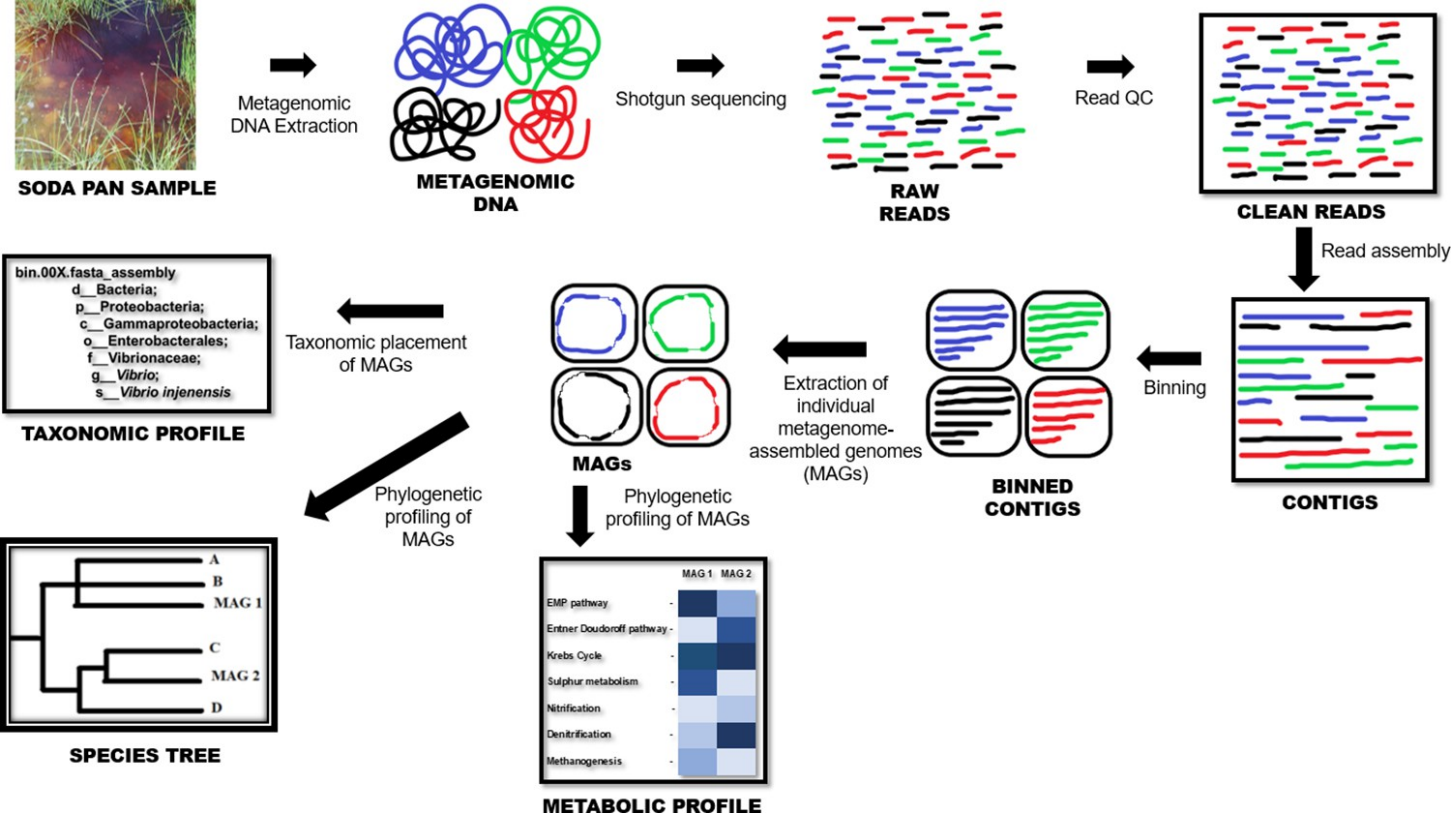
Overview of the sequence analysis workflow employed in this study.

Metagenomic sequence analysis was performed on the United States Department of Energy (DOE) Systems Biology Knowledgebase (KBase) pipeline [16], an integrated sequence analysis platform with several in-built functions that allow for the execution of a number of sequence analysis tasks such as quality assessment, sequence trimming, sequence assembly, binning, and metagenome-assembled genome (MAG) extraction and annotation (Fig 1). Before any analysis, the raw metagenomic DNA reads were quality-checked using the sequence quality tool FastQC (v0.11.9) on the Kbase pipeline using default settings. The quality-checked reads were then passed through the sequence trimming app Trimmomatic (v0.36), with the following parameter settings: leading minimum quality– 3; minimum length– 36; sliding window minimum quality– 15; sliding window size– 4; trailing minimum quality– 3; adapters–null.

#### 2.2.1 Read assembly

Quality-checked metagenomic sequence reads were assembled into contigs using the two de-novo sequence assemblers SPAdes (v3.15.3) and hybridSPAdes (v3.15.3), with all parameters for both assemblers set at default except minimum contig length which was set at 500. The two assemblers perform contig construction based on sequence *k-mer* composition and make use of *de Bruijn* graphs [17,18]. The contig libraries generated were compared in KBase using the "Compare Assembled Contig Distributions" application (v1.1.2), an algorithm that compares contig libraries according to parameters such as number of contigs, maximum contig length, contig length distribution and average contig length. This comparison showed that the hybridSPAdes-generated contig library had marginally relatively better features and was used for the subsequent binning exercise.

#### 2.2.2 Contig binning

The hybridSPAdes-generated contigs were binned using the three binning tools: MaxBin2 (v2.2.4), MetaBat2 (v1.7) and CONCOCT (v1.1). MaxBin2 was run with the following parameter settings: prob_threshold– 0.8; marker set– 107 bacterial marker gene set; minimum contig length– 1000, with the remaining parameters set at default. MetaBat2 was run with all parameters set at default, with minimum contig length of 2500. Finally, parameters for the CONCOCT binning application were set as follows: read mapping tool–Bowtie 2; minimum contig length– 2500; contig split size– 10 000; *kmer* length– 4; maximum number of clusters for VGMM– 400; maximum number of iterations for VGMM– 500. The resultant bin libraries were compared for such features as bin coverage and number of bins generated. The three bin libraries were then optimised using the DAS-Tool (v1.1.2) at default setting, using diamond as the gene identification tool. The DAS tool enables one to generate optimum consensus high-quality bins using, as input, the binning outputs of more than one binning app. In this study, DAS would examine the bins created by the MaxBin2, MetaBat2 and CONCOCT binning tools and come up with a consensus library of high-quality bins from the three bin libraries. Subsequently, the three original bin libraries (MaxBin2, MetaBat2 and CONCOCT) and the DAS tool-generated bin library were compared for coverage and quality and it was noted that the DAS tool-generated bin library had better features, leading to its selection and use for all downstream steps.

#### 2.2.3 Metagenome-Assembled Genome (MAG) extraction and annotation

The DAS tool-generated bin library was firstly checked for completeness and contamination using the CheckM tool (v1.0.18), using default settings. Thereafter, metagenome-assembled genomes (MAGs) were extracted from the DAS bin library using the “Extract Bins as Assemblies from Binned Contigs” application (v1.0.2), at default settings. Extracted MAGs were annotated using the RASTtk tool (v1.073), using default settings. MAG annotation involved determining the size of the genome as well as the detection, identification and functional annotation of open reading frames (ORFs) and genome features such as genes coding for rRNA, tRNA, selenoproteins and pyrrolysoproteins, and clustered regularly interspaced short palindromic repeat (CRISPR) regions present in the MAGs. Additionally, whole-genome maps for selected MAGs were created and annotated using the Proksee web-based application. Proksee allows one to create a complete circular or linear map of bacterial (or other) genomes, and comes with several integrated tools that allow for detailed annotation of the genome maps. In this study, the MAGs were annotated using the following tools: Comprehensive Antibiotic Resistance Database (CARD) Resistance Gene Identifier (RGI) for the identification of antibiotic resistance genes (v1.2.0) [19], CRISPR/ CasFinder (v4.2.20) for the identification of CRISPR/ Cas regions [20], and Prokka (v1.14.6) for the annotation of coding sequences and RNA regions [21].

#### 2.2.4 Taxonomic and phylogenetic profiling of MAGs

Annotated MAGs were taxonomically classified using the GTDB-Tk taxonomic classification tool (v1.7.0) based on the Genome Taxonomy Database (Release202). GTDB R202 is a database of bacterial and archaeal genomic sequences comprising of 254 090 bacterial and 4 316 archaeal genomes (gtdb.ecogenomic.org/stats/r202). The classification was performed using default settings and the following additional settings: minimum alignment percentage– 10; genetic code– 11 (for archaea, most bacteria, most viruses and some mitochondria). The GTDB-Tk tool performs taxonomic classification of the MAGs using single-copy phylogenetic marker sequences to place each genome (MAG) into the GTDB species tree [22]. The MAGs were also inserted into species trees using the “Insert Set of Genomes into Species Tree” app (v2.2.0). This app shows the phylogenetic relationships among the MAGs as well as with other microbes archived in different reference databases.

#### 2.2.5 MAG functional profiling

Apart from taxonomic placement, the MAGs were also subjected to functional profiling using the "Annotate and Distill Assemblies with DRAM" (v0.1.2) application using default settings and the following additional parameters: minimum contig length– 2500; translation table– 11; bit score threshold– 60; reverse search bit score threshold– 350. The DRAM tool searches the MAGs for functional marker genes and groups them into pathways. The pathway completeness for each MAG is determined, making it possible to ascertain likely metabolic roles of each MAG in the microbial community. In this study, DRAM was used to determine pathway completeness for pathways involved in carbon metabolism, nitrogen and sulphur metabolism, electron transport systems and other vital cellular functions. Heatmap plots showing the presence and/ or absence, and completeness of different metabolic pathways and other cellular components were plotted using the “pheatmap” package in R [23]. Additionally, more specific gene family annotation of the MAGs based on Hidden Markov Models (HMMs) was performed using the HMMER program. In this case the dbCAN collection of HMMs built from the Carbohydrate-Active Enzymes (CAZy) was determined using HMMER (v10) at default settings. Summary plots showing metabolic functions and possible ecological roles of the different MAGs were generated. A treemap plot of possible MAG ecological roles was created using the ggplot2 and treemapify packages in R [23].

## 3. Results and discussion

### 3.1 High-quality metagenomic sequence reads were obtained

High quality metagenomic DNA sequences derived from the DNBSEQ^R^ platform were obtained in this study (Table 1).

**Table 1:** Buhera soda pan metagenomic sequence statistics and quality information.

Object Name	Buhera Soda Pan Metagenomic Sequence
Number of Reads	35,876,873
Total Number of Bases	3,587,687,300
Mean Read Length	100.0
Base quality score (range)	34–36
Mean base quality score	35.45
Read quality score (range)	24–38
Mean read quality score	36.0
GC Percentage	52.08%

As shown in Table 1, the sequences obtained were composed of 35.9 million short reads averaging 100 bp in size each. The reads were of high quality, with read quality scores in the range of 24–38. In general, reads with a quality score above 20 are considered to be of high quality and suitable for use in metagenomics studies while reads above quality score 30 are regarded as excellent quality, and reads with quality score below 20 are often trimmed to remove low-quality ends or removed altogether using tools such as Trimmomatic and Cutadapt [24].

### 3.2 HybridSPAdes assembler produced most optimum contig library

Two distinct contig libraries were created using the two de-novo assemblers, SPAdes and hybridSPAdes. However, upon comparison for key contig library features such as contig number, total assembled sequence length, N50 and L50, the hybridSPAdes contig library appeared to have better features (Table 2).

**Table 2:** Features of SPAdes and hybrid SPAdes assembly libraries generated in this study. The values highlighted represent the best output for each parameter.

Contig library feature	ASSEMBLY LIBRARY	
	hybridSPAdes.Assembly	SPAdes.Assembly
Number of contigs	104.964	**100,265**
Number of contigs (> = 10000 bp)	**1,046**	1,030
Number of contigs (> = 100000 bp)	**55**	53
Largest contig (bp)	**312,583**	**312,583**
Total length (bp)	**145,298,474**	136,513,900
Total length (> = 1000 bp)	**96,753,868**	89,478,069
Total length (> = 10000 bp)	**33,554,519**	32.804,010
Total length (> = 100000 bp)	**8,156,189**	7,789,317
N50	**1697**	1675
L50	13884	**12822**

The success of read assembly depends on factors such as the purity of the DNA molecules sequenced, the quality of sequencing output, and sequencing depth. During sequencing, individual reads should sufficiently overlap in a way that makes it possible to form longer consensus sequences during assembly. In this study, the hybridSPAdes assembler managed to assemble a larger proportion of available reads, producing a larger number of contigs greater than 100 kb when compared to SPAdes. In general, a good assembler should produce a small number of large contigs, covering as much of the available sequence reads as possible, and should produce a contig library with high N50 (the length of the shortest contig at 50% of the total assembly when contigs are arranged in descending order of size) and low L50 (the number of contigs, starting with the longest contig, required to create 50% of the total assembly size) values. Based on this broad criterion, hybridSPAdes was adjudged to have been relatively superior, and hence the hybridSPAdes library was used for the subsequent binning step.

### 3.3 Contig binning and bin optimisation

**Fig 2:**
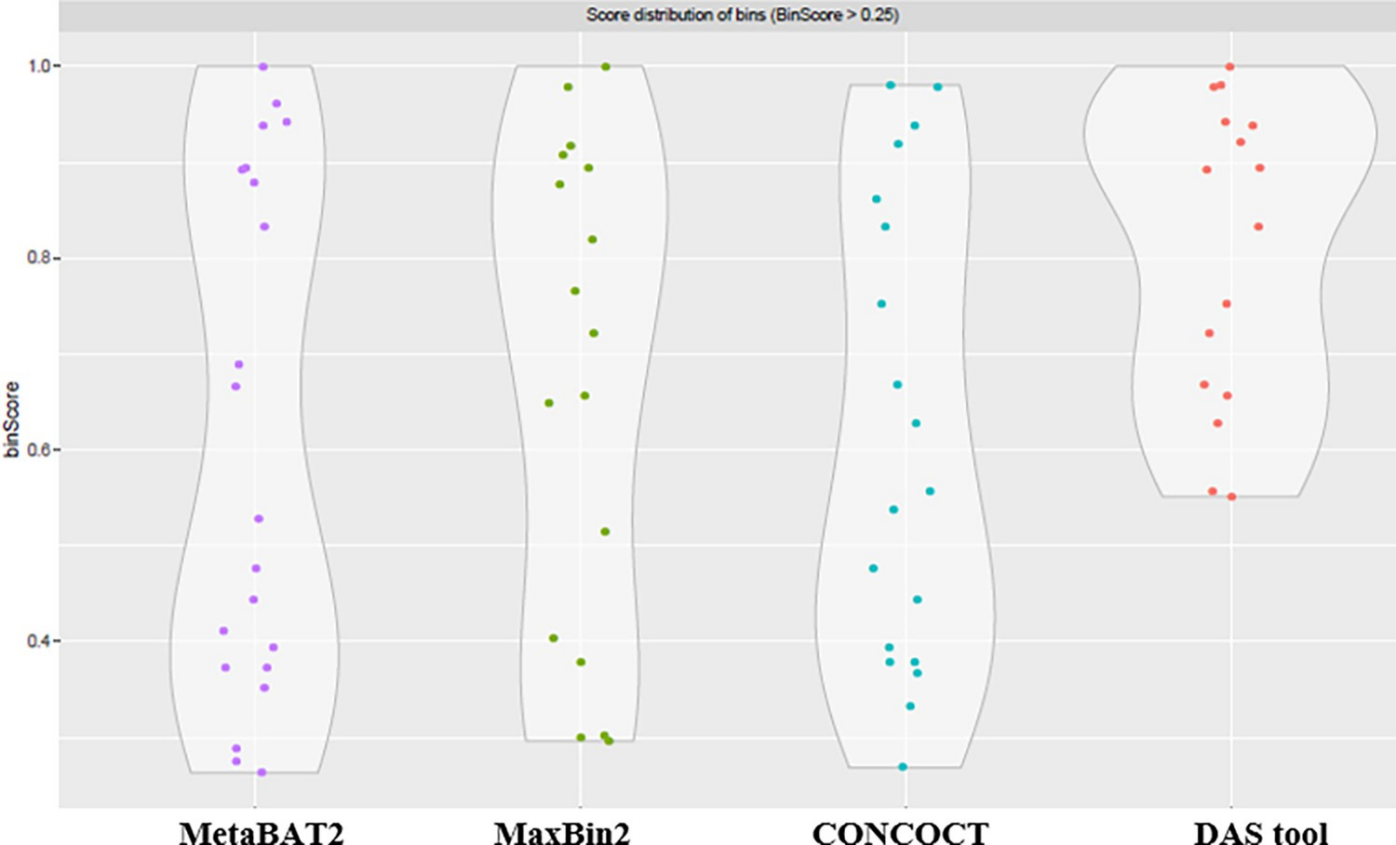
Comparison of the four different bin libraries created in this study. The different coloured dots represent individual bins. Bin library comparison is based on bin score, and only bins with a bin score greater than 0.25 are shown.

The three binning tools used produced different amounts of bins, with CONCOCT producing the most bins at 37, followed by 31 produced by MetaBAT2 and lastly MaxBin2 which produced only 25 bins. Each bin is a collection of contigs that share features such as k-mer profiles and codon usage and, therefore, most likely originate from the same genome. While the three binning tools produced different numbers of bins, further analysis revealed that MaxBin2 managed to place a larger proportion of available contigs (24.81%) into bins as compared to the CONCOCT (6.56%) and MetaBAT2 (5.21%) tools. The ability of the MaxBin2 tool to place more contigs into bins will likely produce bins with a higher degree of completeness, which usually leads to the generation of higher quality MAGs. Another key observation is that even though MaxBin2 managed to place only 24.81% of the 100 265 contigs available into bins, the total length of these binned contigs amounts to more than half (55.4%) of the assembled sequence length. This further highlights the superiority of the MaxBin2 binning tool in the specific context of this study. The MaxBin2 bin library was therefore determined to be the best of the three bin libraries. Additionally, bin optimisation using the DAS tool created a consensus bin library composed of 16 bins using, as input, the output of the MaxBin2, MetaBAT2 and CONCOCT tools. A comparison of these 4 bin libraries clearly showed the superiority of the DAS bin library over the other three (Fig 2).

As shown in Fig 2, the DAS bin optimisation tool assembled a library of bins of relatively higher quality by consolidating bins from the other binning apps. In this case, DAS selected only bins with a minimum bin score of 0.5. The DAS bin library represents a consolidation of high-quality bins from the other binning app, and as a consequence was used for MAG extraction.

### 3.4 Sixteen medium-to-high-quality MAGs were recovered from the Buhera soda pans

**Fig 3:**
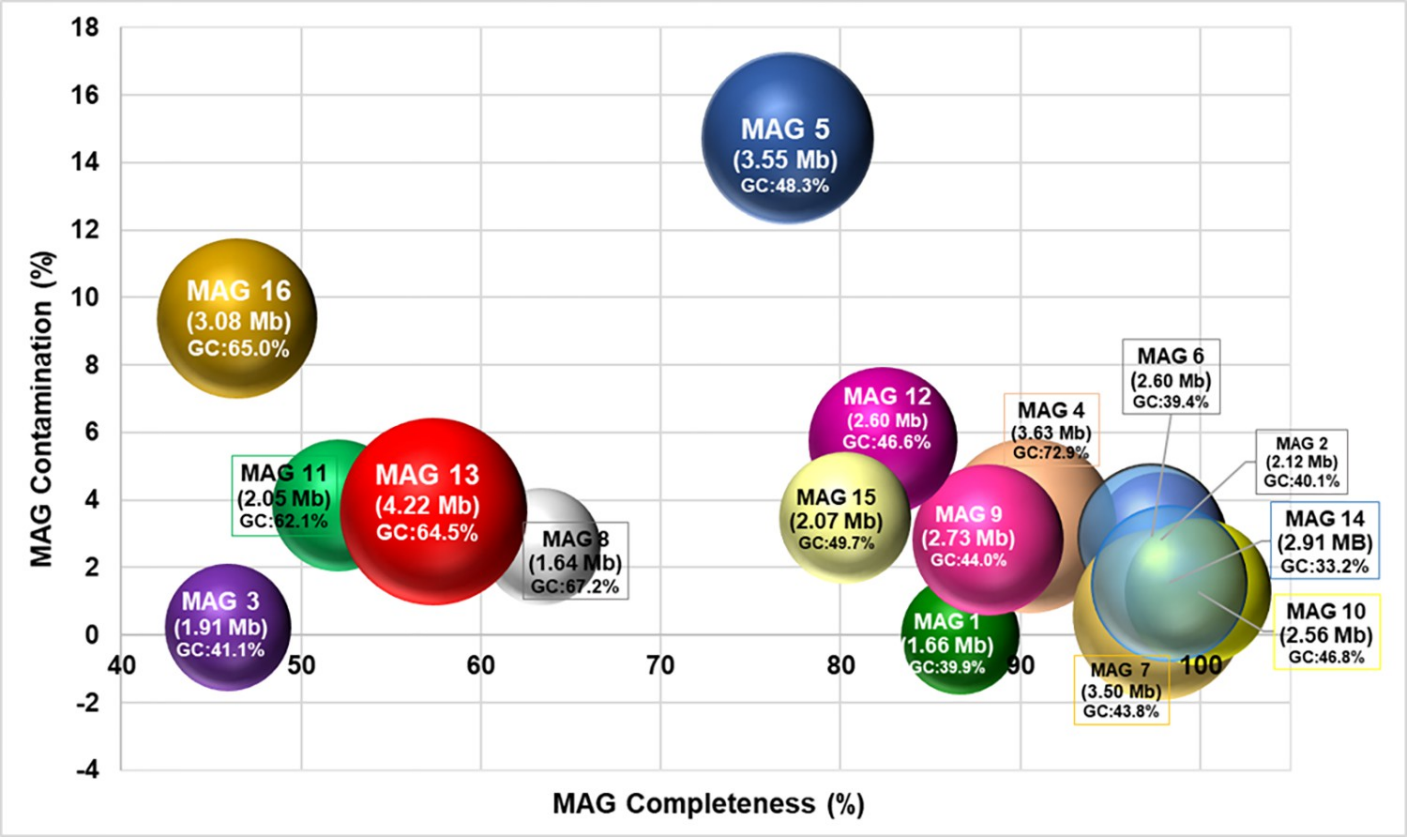
MAG size and level of completeness and contamination. Completeness and contamination were assessed using the CheckM tool (v1.0.18), and MAG size determination and MAG annotation were done using the RASTtk tool (v1.073). Each bubble represents a MAG. The position of a bubble shows the level of completeness (horizontal axis) and contamination of a MAG (vertical axis), while the size of each bubble shows MAG size (in nucleotides). MAG size and G+C content is inscribed on each bubble.

CheckM quality assessment showed that a majority of the MAGs possessed high levels of completeness and low levels of contamination (Fig 3).

Of the sixteen MAGs assessed, a total of 11 (69%) were characterised by a level of completeness above 70%, and 14 (88%) had contamination levels of 6% or lower. Based on the criteria used by the CheckM tool, 31% of the MAGs produced in this study were classified as “high-quality draft” MAGs, 63% were “medium-quality draft” MAGs, whereas only A]a single MAG (MAG 3) was of low quality with a completeness of 45.95% [25]. MAG completeness and level of contamination are important parameters used to describe MAG quality, where completeness roughly measures the fraction of a microbial genome captured in a MAG and contamination measures the amount of foreign sequences that may be present in a MAG. The medium- and high-quality MAGs obtained in this study meet the minimum requirements for "Minimum Information on Metagenome-assembled Genomes" (MIMAGs) as proposed by the Genomics Standards Consortium [25]. Overall, the quality of most MAGs obtained in this study is suitable for further downstream analysis of the genomes obtained.

The 16 MAGs obtained in this study were highly diverse in terms of size (Fig 3) and organisation, as exemplified by the differential distribution of genome features such as coding and non-coding genes, non-coding repeats and RNA-coding genes. The GC content of the MAGs ranged from a low of 33.20% (MAG 14) to a high of 72.90% (MAG 4).

The largest MAG created in this study (MAG 13) was 4,415,129 bp in size. Four other MAGs (MAG 4, MAG 5, MAG 7 and MAG 16) exceeded 3 Mb in size. These MAGs are within range of the size of the average bacterial genome. Though bacterial genome size varies considerably, it is generally accepted that the average bacterial genome is about 5 Mb in size and encodes approximately 5000 proteins [26]. The closer a MAG is to an actual genome in terms of size, the more information one will get of its genetic elements and metabolic potential upon analysis. This enables one to get more insight about the microbial inhabitants of an environment and their possible ecological roles in the ecosystem. The annotation tool used to annotate genome features of the MAGs in this study, RASTtk, calls open reading frames using both Prodigal [27] and Glimmer3 [28]. RAST uses the k-mer approach to find genes in reference databases that are homologous to the query sequences, using a collection of gene family functional annotations to rapidly annotate any matches [14].

### 3.5 All recovered MAGs belong to only five phyla under domain bacteria

All the MAGs recovered from the Buhera soda pans were shown to belong to domain bacteria, all being distributed among the five phyla *Pseudomonadota*, *Bacillota*, *Chloroflexota*, *Bacteroidota* and *Deinococcota* (Table 3).

**Table 3:** Taxonomic placement of MAGs. The taxonomic assignment as well as the identity of closest match from reference databases, where available, is shown. MAGs are clustered according to phylum shown in different colour blocks.

MAG	TAXONOMIC CLASS			
	Phylum	Class	Genus	Species
MAG 1	*Bacillota*	*Bacilli*	*-*	*-*
MAG 2			*UBA2227*	*UBA2227*
MAG 6			***Alkalibacterium***	*-*
MAG 9		*Clostridia*	*Proteiniclasticum*	*-*
MAG 14			*Acetoanaerobium*	*-*
MAG 15		*Dethiobacteria*	*UBA993*	*-*
MAG 7	*Pseudomonadota*	*Gamma-proteobacteria*	***Vibrio***	*Vibrio injenensis*
MAG 12			***Nitrincola***	*-*
MAG 16			***Thioalkalivibrio***	*-*
MAG 8		*Alpha- proteobacteria*	*Sandaracinobacter*	*-*
MAG 11			*Erythrobacter*	*-*
MAG 5	*Chloroflexota*	*Anaerolineae*	*Brevefilum*	*-*
MAG 13			*JAAEKA01*	*-*
MAG 3	*Bacteroidota*	*Bacteroidia*	***Cecembia***	-
MAG 4	*Deinococcota*	*Deinococci*	*JAABTL01*	*-*
MAG 10	*Campylobacterota*	*Epsilon-proteobacteria*	*UBA1877*	*-*

Two bacterial phyla, *Pseudomonadota* and *Bacillota*, accounted for 69%% of all MAGs created, indicating their dominance in the Buhera soda pans. This observation is consistent with our findings in an earlier study where read-based taxonomic analysis showed that *Pseudomonadota* and *Bacillota* constituted 67% of the Buhera soda pan microbiome [1]. Several studies have also reported on the dominance of phylum *Psedomonadota* and *Bacillota* in soda pans and lakes [2,3,29]. Additionally, ten out of the sixteen MAGs were classified up to genus level, with a notable finding being the presence of the following halophilic/ haloalkaliphilic genera: *Alkalibacterium*, *Vibrio*, *Thioalkalivibrio*, *Cecembia* and *Nitrincola* being. These halophiles/ haloalkaliphilic genera are commonly associated with saline and saline-alkaline environments, thus their presence in abundance in the Buhera soda pans highlights its status as a natural habitat for salt and alkali-loving extremophiles. Only two MAGs could be classified at species level, suggesting the possible presence of previously uncharacterised microorganisms in this habitat. This finding calls for further research with a view to full describe these potentially novel organisms in terms of their morphology, genomics and physiology, and a possible design of protocols for their cultivation.

**Fig 4:**
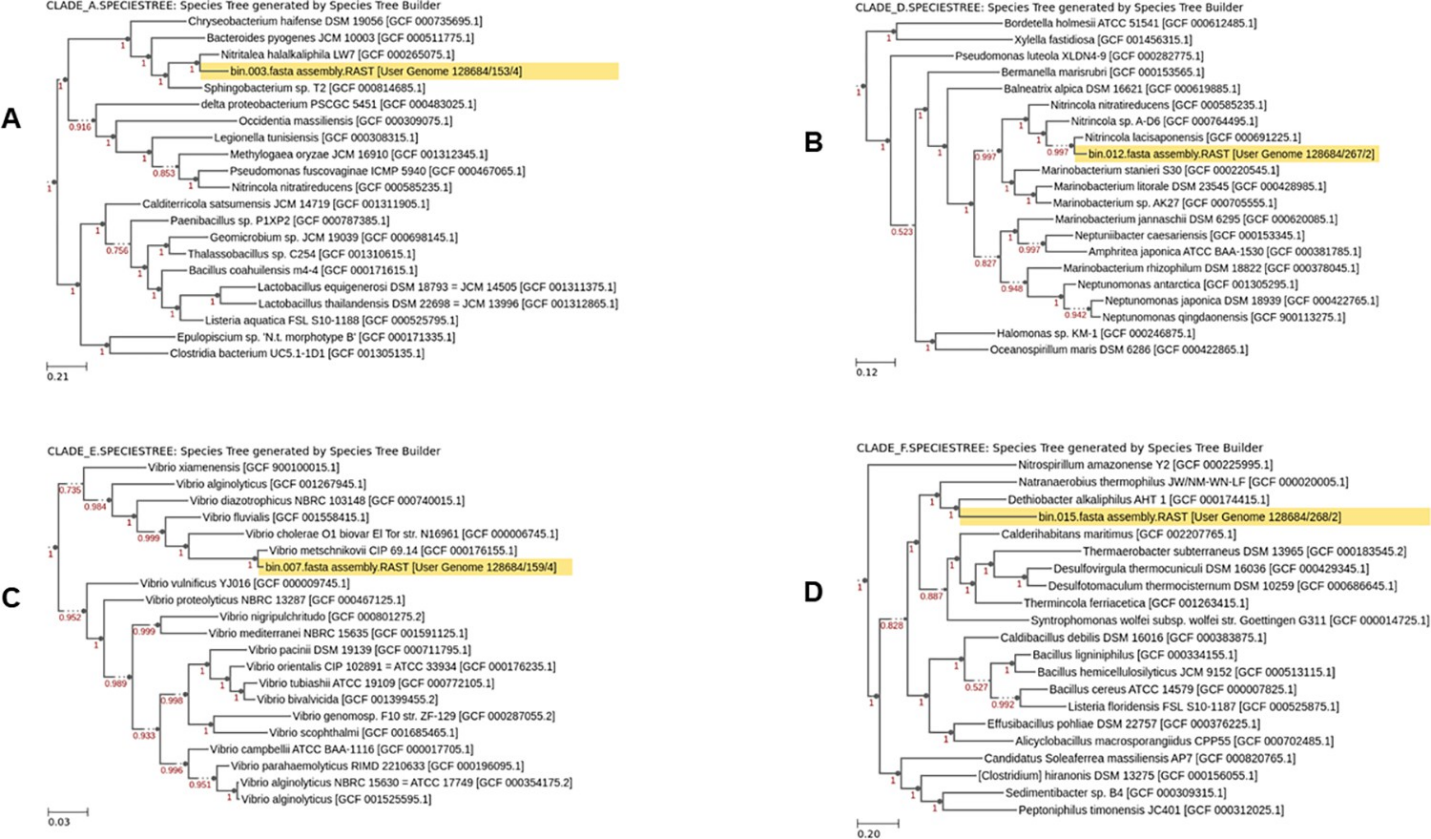
Phylogenetic placement of selected MAGs with proximal reference genomes from RefSeq. Four MAGs are shown each in its own phylogenetic tree; A: Bin 003 (MAG 3), B: Bin 012 (MAG 12), C: Bin 007 (MAG 7), and D: Bin 015 (MAG 15). Scale bars show branch length distance that approximates the amino acid substitution distances between the genomes in the tree, as measured from the protein multiple sequence alignments of the single-copy universal COGs found in the genomes. Red values at nodes indicate the FastTree-261 local support values for placing a split at that node, with 1 showing maximal support in the topological placement.

Additionally, all MAGs were placed into individual species trees. Select MAGs are shown in Fig 4.

The 4 MAGs shown appeared to cluster closely with related organisms in the different species trees (Fig 4). MAG 3 clustered closely with *Nitritalea halalkaliphila* LW7, an alkaliphile isolated from Lonar Lake in Maharastra, India, which has an optimum pH for growth of 10.0–10.5 and optimum salinity of 2300–6500 mg/l [30]. Similarly, MAG 007 was shown to cluster closely with members of the *Vibrio* genus and is in all likelihood a member of this genus as well. In fact MAG 7 was taxonomically identified as *Vibrio injenensis*. Bacteria belonging to the *Vibrio* genus have been commonly isolated from marine as well as saline and saline-alkaline aquatic environments [31]. In previous work, we discovered that the *Vibrio* is the most abundant bacterial genus in the Buhera soda pans [1]. Meanwhile, MAG 12 clustered closely to *Nitrincola lacisaponensis*, and appears to belong to the same genus (Fig 4). Genus *Nitrincola* is composed of different haloalkaliphilic bacterial species belonging to family *Oceanospirillaceae*, with several of its members having been isolated from soda pans and lakes [32,33]. Lastly, MAG 15 clustered alongside *Dethiobacter alkaliphus* AHT 1 in the phylogenetic tree. *Dethiobacter* is an obligately haloalkaliphilic, facultatively chemolithoautotrophic, sulphidogenic, rod-shaped and anaerobic bacterium isolated from soda lake sediments and capable of growing through respiring sulphur and thiosulphate [4].

Bacterial lineages that are relatively more abundant in a microbial community are more likely to be recovered as high-quality MAGs from short-read metagenomic sequences compared to those that are less prevalent [14]. The greater relative abundance of these dominant lineages in the microbial community ensures sufficient read representation in the sequencing libraries, making it possible to assemble more complete genomes of these particular lineages. Due to their higher relative abundance, these leading lineages are likely to play more dominant roles in the microbiome, including contributing a larger proportion of the biomass and energy flowing through the microbiome [14]. The recovery of several halophilic/ haloalkaliphilic MAGs in the Buhera soda pans suggests their abundance in this environment, and may point to them playing dominant ecological roles in this ecosystem. In fact, functional analysis showed that the recovered MAGs belong to taxa that are likely involved in important geochemical processes such as the carbon, nitrogen and sulphur cycle.

### 3.6 Functional profiling reveal possible ecological roles of MAGs

**Fig 5:**
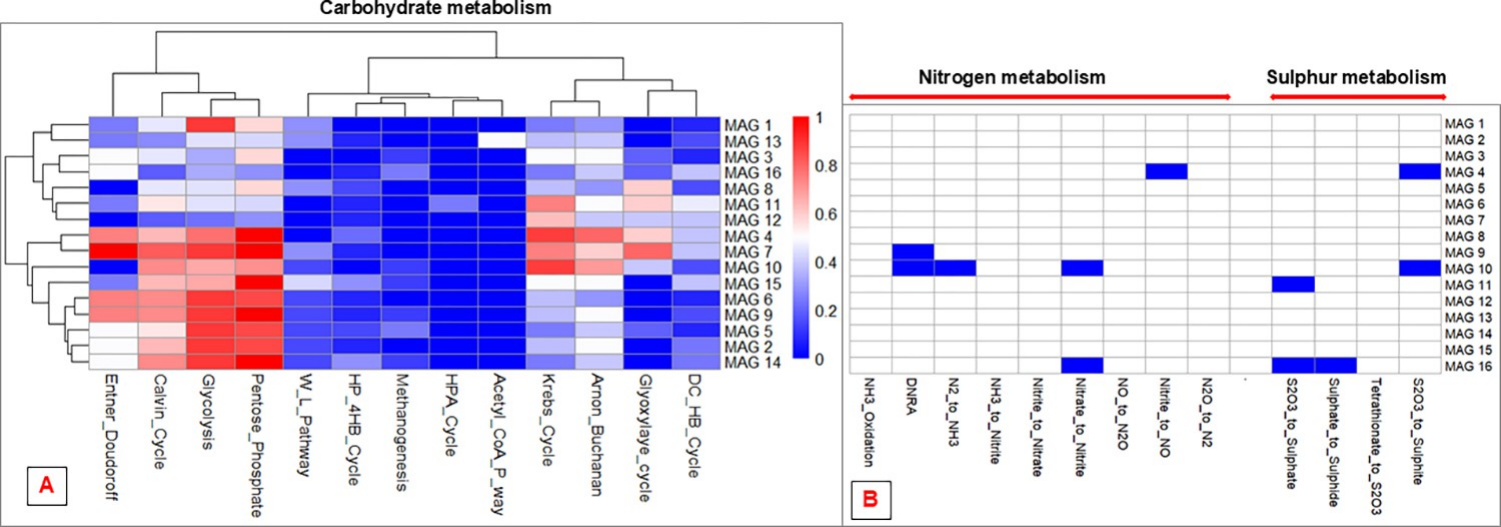
Heatmap plots showing the presence/ absence and level of completeness of metabolic pathways involved in carbohydrate, nitrogen and sulphur metabolism among the MAGs. MAGs are shown as rows and the various metabolic pathways as columns. A: Carbohydrate-metabolising pathway presence and completeness. Cell colour intensity indicates the fraction of functional marker genes (% completeness) for the pathway that are present in a MAG, with red and blue cell colour showing higher and lower levels of pathway completeness, respectively. Full names for some pathways shown in the plot in short form are as follows: W-L pathway -> Reductive Acetyl-CoA pathway (Wood-Ljungdahl pathway), HP-4HB cycle -> Hydroxypropionate-hydroxybutylate cycle, HPA cycle -> 3-Hydroxypropionate bi-cycle, DC-HB cycle -> Dicarboxylate-hydroxybutyrate cycle. Clustering of pathways as well as of MAGs in shown. B: Presence or absence of pathways involved in nitrogen and sulphur metabolism. A shaded cell shows that the pathway is present whereas an empty cell denotes an absent pathway. The full name for the pathway shown as DNRA on the plot is dissimilatory nitrite reduction to ammonia.

Analysing the functional profiles of the MAGs, one gets greater insight into individual organisms’ mode of life, their ecological roles in the microbial community as well as major biological processes that are possibly active within the environment. For instance, functional analysis revealed that the MAGs recovered in this study possess a wide array of carbohydrate-metabolising pathways, with most MAGs appearing to possess more than one carbohydrate-metabolising pathway (Fig 5A).

Based on pathway completeness, it appeared that a majority of the MAGs have a preference for oxidizing carbohydrates using one or more of four pathways, namely the Embden-Meyerhof-Parnas (EMP) pathway, Pentose phosphate pathway, Entner-Doudoroff pathway and Tricarboxylic Acid (Krebs) Cycle (Fig 5A). These pathways are generally used to oxidise carbohydrates, generating electrons that are used in ATP synthesis as well as various organic intermediates that are used in cellular biosynthesis. Several microorganisms have the ability to switch between different carbohydrate-oxidising pathways in response to changes in substrate availability or other environmental stimulus [34]. The ability of microorganisms to switch between different ATP-generating, carbohydrate-oxidising pathways ensures survival in the face of stressful conditions such as substrate depletion or the displacement of microorganisms to new environments with different carbon sources. The presence of genetic elements for the Calvin cycle in some MAGs suggest an autotrophic mode of carbon acquisition by these MAGs. One key finding is the low prevalence of pathways involved in bacterial carbon fixation such as the Wood-Ljungdahl (WL) pathway, the 3-hydroxypropionate/4-hydroxybutyrate cycle, methanogenesis, Acetyl-CoA pathway and the 3-Hydroxypropionate bi-cycle. These pathways are involved in carbon fixation in different microorganisms, and their paucity among the MAGs may point to a less important role that carbon fixation (and bacterial autotrophy) plays in the Buhera soda pan microbial community. Further analysis showed evidence of the use of different electron transport complexes by the Buhera soda pan microbial community. This, coupled by the presence of various oxidative processes such as the EMP pathway, Entner Doudoroff pathway and the TCA cycle, points to the importance of respiration as an energy-generating process among the Buhera soda pan microorganisms.

Additionally, some MAGs were shown to possess pathways involved in some key aspects of the nitrogen cycle. Only one MAG, MAG 10, was shown to possess the ability to fix nitrogen to ammonia. MAG 10 was taxonomically classified as an unclassified *Campylobacteraceae*. MAG 10 was also shown to possess pathways for the dissimilatory reduction of nitrite to ammonia and to be capable of reducing nitrate to nitrite, two functions that it shared with MAG 9 (*Proteiniclasticum* sp.) and MAG 16 (*Thioalkalivibrio*), respectively. Thus MAG 10 appears to be an important player in the nitrogen cycle in the Buhera soda pan microbial community. Genome analysis revealed that MAG 10 possesses *nifD*, *nifK* and *nifH* genes which encode different components of nitrogenase, the enzyme responsible for bacterial biological nitrogen fixation. The *nifD* and *nifK* genes encode the heterotetrameric core of bacterial nitrogenase, while *nifH* encodes the dinitrogenase reductase subunit of this enzyme [35].

Apart from their involvement in some components of the nitrogen cycle, some MAGs were also shown to possess pathways essential in key aspects of the sulphur cycle. For example, MAG 16 (*Thioalkalivibrio*) was shown to possess pathways for the oxidation of thiosulphate to sulphate by the sulphur oxidising (SOX) complex, a function it shared with MAG 11 (*Erythrobacter* sp.), as well as the dissimilatory reduction of sulphate to sulphide. Two more MAGs, MAG 4 (an unclassified *Deinococcota*) and MAG 10, were shown to be capable of converting thiosulphate to sulphite. MAG 16 (*Thioalkalivibrio*) appeared to be an important player in the Buhera soda pan sulphur cycle. The *Thioalkalivibrio* are an important genus of chemolithoautotrophic bacteria that use energy derived from the oxidation of sulphide, thiosulphate, sulphate, elemental sulphur and polysulphides to fix CO_2_ to sugars via the Calvin cycle [36,37]. This genus often thrives in saline and alkaline environments such as soda pans and lakes and is an important player in the sulphur cycle in these ecosystems [37,38].

**Fig 6:**
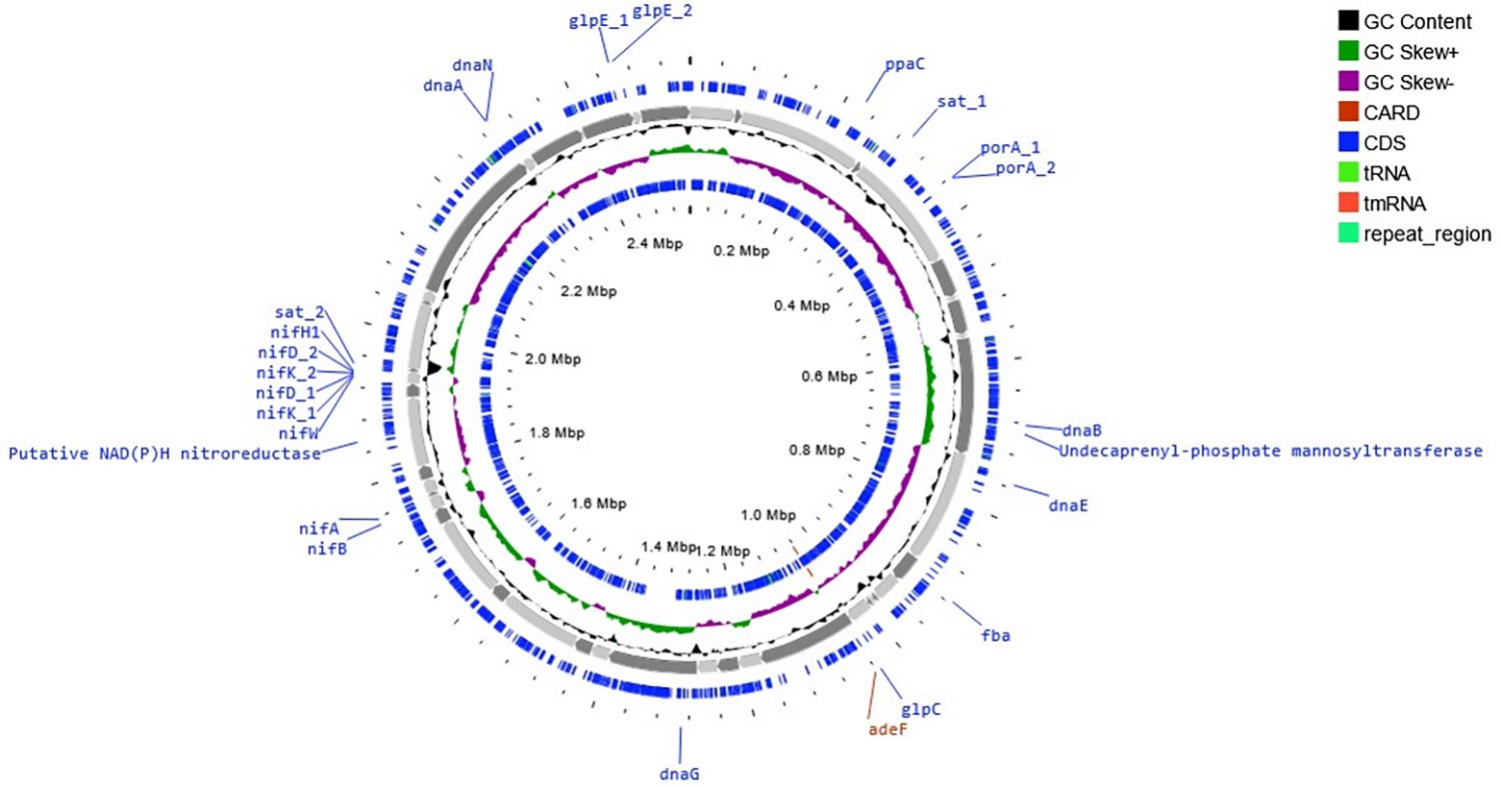
Whole genome map of MAG 010 (unclassified *Campylobacteraceae*). From outside to inside: Coding sequences (CDS) located on the forward strand (blue), sequence backbone made up of 46 contigs joined end-to-end (grey), GC content (black), GC skew (purple when GC skew < 0, or olive when GC skew > 0), coding sequences (cds) located on the reverse strand (blue).

Proksee, a web-based platform, was used to construct the circular genome map of MAG 10 and show the spatial organisation of this genome and its different functional sequences (Fig 6).

As shown, MAG 010 possesses two clusters of genes (possibly operons) supporting nitrogen fixation. The first cluster contains the *nifA* and *nifB* genes and is located on the reverse strand of contig 29 (NODE_476_length_21671_cov_6.024796) in the MAG map, and the second cluster, located on the reverse strand of contig 35 (NODE_549_length_18964_cov_5.559099), contains the *nifD*, *nifH*, *nifK* and *nifW* genes. The *nifA* gene encodes a regulator protein, which when expressed (under conditions of low fixed nitrogen availability) activates the transcription of the rest of the *nif* gene complex, leading to their expression and eventual nitrogen fixation. In addition to the nitrogen fixation complex, genes involved in sulphur reduction can also be located on the map, for example the *sat1* and *sat2* genes which encode the enzyme sulphate adenyltransferase (EC 2.7.7.4) which catalyses the adenylation of sulphate using ATP to synthesise adenylyl sulphate and pyrophosphate, the latter which can be hydrolysed by the *ppaC* (pyrophosphatase) gene which was also located on the forward strand of contig 11 (NODE_11_length_182382_cov_6.080246). These enzymes play a key function in both assimilatory sulphur reduction and dissimilatory sulphur oxidation, and are hence key to the sulphur cycle. The presence of the *fba* gene which encodes the enzyme Fructose 1,6 bisphosphate aldolase suggests a possible mechanism of carbohydrate metabolism through the glycolytic pathway by this MAG. Other genes found on the map include an antibiotic resistance gene *adeF* which encodes a drug efflux pump, and genes encoding some subunits of the DNA polymerase enzyme (*dnaA*, *dnaB*, *dnaE*, *dnaG* and *dnaN*) involved in DNA replication.

**Fig 7:**
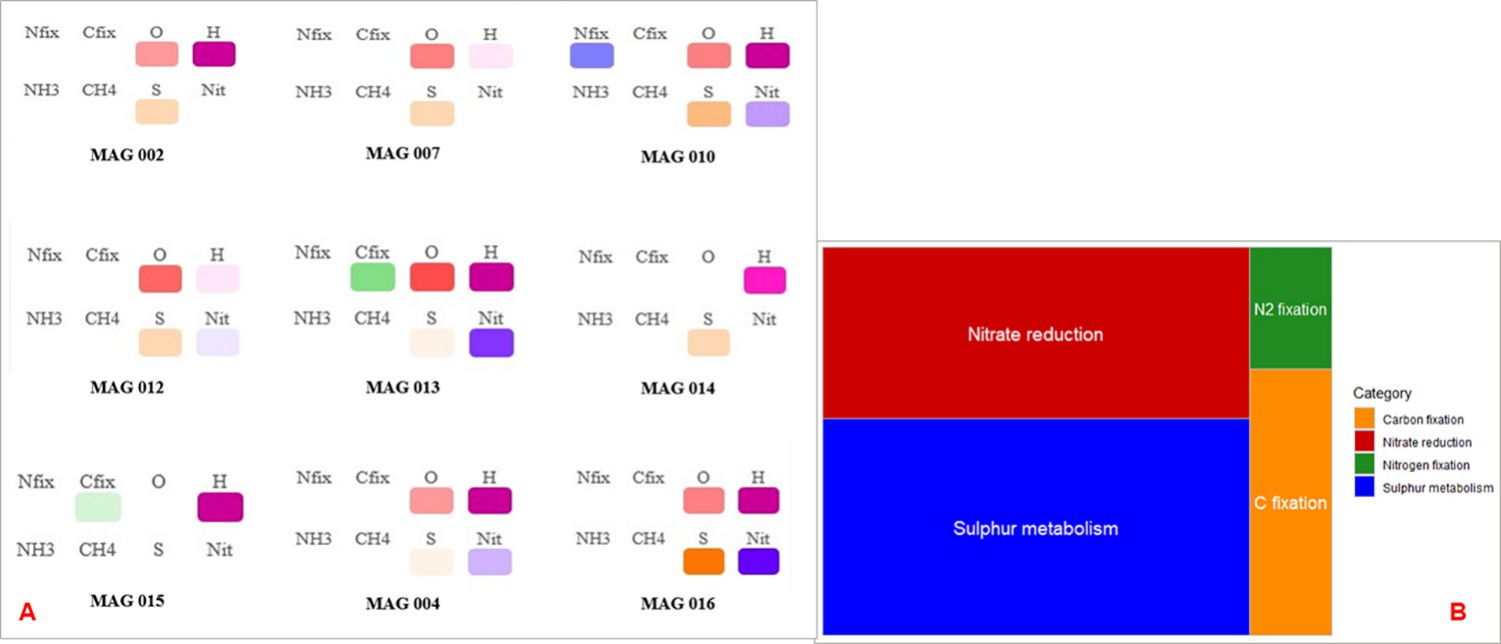
Summary of Buhera soda pan MAG library functional annotation. A: Prevalence of genes for enzyme families involved in key biogeochemical transformations found using MicroTrait [39]. Bioprocess categories are presented using different colours as follows: Nitrogen-fixing genes (blue), carbon-fixing genes (green), oxygen-interacting genes (red), hydrogenases (magenta), ammonia-interacting genes (light blue), methane cycle genes (teal), sulfur and sulfate redox genes (orange) and nitrate redox genes (blue). A more intense box colour indicates a larger number of genes found in that category, whereas an empty space means no genes were found for that metabolic pathway. B: TreeMap plot showing the levels of major biogeochemical processes in the Buhera soda pans. The size of each nested rectangle is proportional to the level of that geochemical process in the microbial community.

The possible roles of selected MAGs in a number of different biogeochemical pathways were summarised as shown in Fig 7.

The summary plots show the involvement of the MAGs in different aspects of the carbon, hydrogen, nitrogen and nitrogen cycles (Fig 5A). The majority of MAGs were found to possess genes belonging to pathways for participation in two major processes, sulphur and sulphate metabolism and nitrate reduction processes (Fig 5B). The greater prevalence of sulphur and sulphate metabolism supports the hypothesis that a significant proportion of organisms in this ecosystem respire anaerobically using sulphur and/ or sulphate as terminal electron acceptors. These findings are supported by our earlier observations showing the dominance of sulphur compounds in the Buhera soda pans. Low levels of carbon and nitrogen fixation were noted (Fig 5B), an observation supported by the low prevalence of pathways involved in carbon fixation among the MAGs (Fig 5A). albeit at different levels.

**Fig 8:**
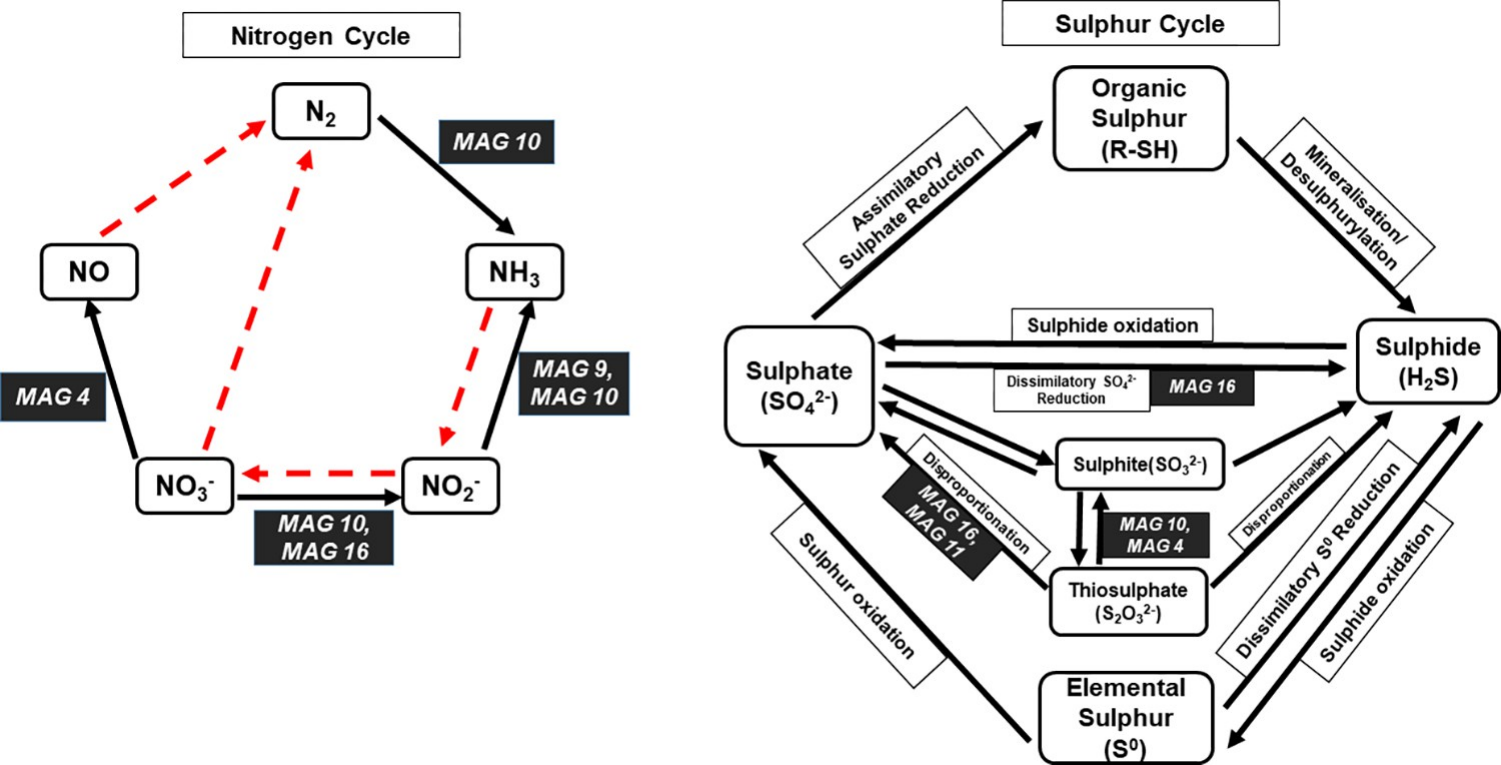
Roles of different MAGs in the nitrogen and sulphur cycles. MAGs possessing genes for specific pathways are labeled on the cycles.

Only 2 of the 16 MAGs appeared to be highly likely autotrophic, with one being a photoautotroph (MAG 13) and the other one a chemolithoautotroph (MAG 15). MAG 13 was classified under the unclassified bacterial class *Anaerolineae* under the metabolically-diverse bacterial phylum *Chloroflexota*¸ while MAG 15 was shown to be likely an unclassified *Dethiobacteria* under phylum *Bacillota*. If indeed true, the presence of primary producers in the ecosystem is important as they contribute to the production of organic matter required by the heterotrophic fraction of the soda pan microbiome. In summary, the possible roles of the different MAGs in the nitrogen and sulphur cycles are shown in Fig 8.

Five out of the sixteen MAGs recovered from the Buhera soda pans were shown to possess pathways for their involvement in four steps of the nitrogen cycle (Fig 8). MAG 10 (unclassified *Campylobacteraceae*) possessed pathways for the fixation of nitrogen to ammonia, and the reduction of nitrate to nitrite and nitrite to ammonia. Two more MAGs, that is MAG 16 and MAG 9, were also shown to possess pathways for the reduction of nitrate to nitrite and nitrite to ammonia, respectively. Several microorganisms, known as nitrate-reducing bacteria, are capable of reducing nitrate, as an electron acceptor, to nitrite and then to nitrogen gas or ammonia and are important in the nitrogen cycle of aquatic environments [40]. Only one MAG (MAG 4) was shown to have genes for the reduction of nitrate to nitric oxide (NO), perhaps as part of a pathway for the denitrification of nitrate.

Pathways for the dissimilatory reduction and oxidation of different sulphur compounds were observed in five of the sixteen MAGs. These include the pathway for the dissimilatory reduction of sulphate to sulphide observed in MAG 16 (*Thioalkalivibrio* sp.). Dissimilatory sulphate reduction occurs when sulphate-reducing bacteria generate energy through anaerobic respiration, using sulphate as a terminal electron acceptor and producing hydrogen sulphide as a bi-product [41]. The pathway for the direct oxidation of thiosulphate to sulphate without intermediates, a pathway commonly found in *Alphaproteobacteria* [42], was identified in MAG 11 (*Erythrobacter* sp., an *Alphaproteobacteria*) and MAG 16 (a *Gammaproteobacteria*). On the contrary, MAG 4 and MAG 10 were shown to possess the pathway for the oxidation of thiosulphate to sulphite, an intermediate in the oxidation of thiosulphate to sulphate. The oxidation of thiosulphate appeared to be an important process in the bioenergetics of the sulphur bacteria of the Buhera soda pans. The oxidation of thiosulphate to sulphate occurs with or without the generation of intermediates as part of the disproportionation of thiosulphate in members of the *Pseudomonadota* phylum [42]. Sulphur disproportionation is a dissimilatory energy-generating process whereby sulphur species such as elemental sulphur, sulphite or thiosulphate act as both an electron donor and acceptor, resulting in the production of sulphate and sulphide [41]. In general, the oxidation of different sulphur species provides electrons required for energy generation by different groups of sulphur bacteria.

## 4. Conclusion

In this study, we report on the recovery and annotation of 16 microbial MAGs, all belonging to five phyla within the bacteria domain, from a previously unexplored and uncharacterised extreme natural environment. Six of the reconstructed MAGs could not match any cultured reference strains, suggesting that the Buhera soda pans may harbour novel and previously uncultured bacteria. Most of the MAGs were of medium-to-high-quality, harbouring sufficient genetic information to enable their detailed annotation. Five of the sixteen MAGs belonged to halophilic/ haloalkaliphilic genera, suggesting a possible dominance of these salt- and alkali-loving extremophiles in the Buhera soda pans. Functional annotation of the MAGs revealed a wide array of metabolic pathways among the MAGs, with key processes being dissimilatory oxidation and reduction of sulphur, nitrate reduction and carbon and nitrogen fixation. More work is needed in order to allow us to learn more about the composition and biotechnological potential of this complex microbial habitat.
